# Comment on ‘Pre-treatment levels of circulating free IGF-1 identify NSCLC patients who derive clinical benefit from figitumumab’

**DOI:** 10.1038/bjc.2011.412

**Published:** 2011-10-11

**Authors:** H Shimokawa, H Uramoto, F Tanaka

**Affiliations:** 1 Second Department of Surgery, School of Medicine, University of Occupational and Environmental Health, 1-1 Iseigaoka, Yahatanishi-ku, Kitakyushu 807-8555, Japan

**Sir**,

We read with great interest the online article by Gualberto *et al* (2011) and congratulate the authors on completing this randomized phase II trial. This study concludes that free IGF-1 (fIGF-1) may contribute to the identification of a subset of NSCLC patients who would benefit from figitumumab therapy. They also showed that the plasma fIGF-1 levels were found to be associated with tumour vimentin expression (*P*=0.021) and are inversely associated with E-cadherin (*P*=0.152) in 45 out of 110 NSCLC patients. Therefore, investigation to verify whether there is a correlation in the appearance of the ligand and receptor in pretreatment samples was warranted, as the IGF-1 receptor (IGF-1R) expression was tightly regulated at multiple levels (Chitnis *et al*, 2008).

We have recently investigated the expression of EMT-related molecules (Yamashita *et al*, 2010) and IGF-1R (Nakagawa *et al*, 2011) in NSCLC patients who underwent curative surgery. We have analysed the correlation between EMT molecules and IGF-1R. In our study, tumour specimens were collected from 182 consecutive patients who underwent a complete resection for lung adenocarcinoma from 2003 to 2007 in our department. According to the pathological stage, 105 patients had tumours of stage IA, 39 IB, 13 IIA, 6 IIB, and 16 IIIA, and 3 patients had tumours of stage IIIB, as classified according to the new TMN (seventh edition) classification for lung cancer. We analysed the associations between the expression levels of IGF-1R, E-cadherin, *γ*-catenin, and vimentin in the primary lung adenocarcinoma by immunohistochemisty.

Positive expression of IGF-1R was detected in 43 (23.6%) of the 182 cases. The expression of E-cadherin, *γ*-catenin, and vimentin was detected in 94 (51.4%), 82 (40.4%), and 32 (17.5%) patients, respectively. The *χ*^2^-test demonstrated a significant association between the IGF-1R and E-cadherin expression (*P*=0.009) and the *γ*-catenin expression (*P*=0.020). In contrast, IGF-1R failed to show any association with vimentin (*P*=0.840). Furthermore, the multivariate logistic regression models indicated that the expression of E-cadherin was an independent predictor for positive IGF-1R expression (*P*=0.026), and *γ*-catenin had a borderline correlation (*P*=0.058). Therefore, there might be a negative feedback loop between these molecules (Baserga, 2007) or sophisticated changes in the physiological concentration of IGF-1R (Pollak, 2008) in the IGF/IGF-1R axis. In fact, IGF-1R-dependent adhesion was restored when both E-cadherin and IGF-1R were co-expressed in breast cancer cell *in vitro* (Mauro *et al*, 2001).

The IGF-1R pathway has also been shown to exhibit cross-talk with a number of other signalling pathways (Zha and Lackner, 2010). There were differences in the patient selection with regard to patients not to amenable to curative treatment between Gualberto’s report and the curative subgroups in our analysis. A detailed examination of a prospective trial can produce new information about the relationships between IGF/IGF-1R and the EMT, and not only the IGF-related molecules, but also the EMT status, may be predictive for the response to IGF-1R-inhibitors.

## References

[bib1] Baserga R (2007) Is cell size important? Cell Cycle 6: 814–816 17404503 10.4161/cc.6.7.4049

[bib2] Chitnis MM, Yuen JS, Protheroe AS, Pollak M, Macaulay VM (2008) The type 1 insulin-like growth factor receptor pathway. Clin Cancer Res 14: 6364–6370 18927274 10.1158/1078-0432.CCR-07-4879

[bib3] Gualberto A, Hixon ML, Karp DD, Li D, Green S, Dolled-Filhart M, Paz-Ares LG, Novello S, Blakely J, Langer CJ, Pollak MN (2011) Pre-treatment levels of circulating free IGF-1 identify NSCLC patients who derive clinical benefit from figitumumab. Br J Cancer 104: 68–74 21102589 10.1038/sj.bjc.6605972 PMC3039819

[bib4] Mauro L, Bartucci M, Morelli C, Andò S, Surmacz E (2001) IGF-I receptor-induced cell-cell adhesion of MCF-7 breast cancer cells requires the expression of junction protein ZO-1. J Biol Chem 276: 39892–39897 11518717 10.1074/jbc.M106673200

[bib5] Nakagawa M, Uramoto H, Shimokawa H, Onitsuka T, Hanagiri T, Tanaka F (2011) Insulin-like growth factor receptor-1 expression predicts postoperative recurrence in adenocarcinoma of the lung. Exp Ther Med 2: 585–590 22977544 10.3892/etm.2011.258 PMC3440752

[bib6] Pollak M (2008) Insulin and insulin-like growth factor signalling in neoplasia. Nat Rev Cancer 8: 915–928 19029956 10.1038/nrc2536

[bib7] Yamashita T, Uramoto H, Onitsuka T, Ono K, Baba T, So T, So T, Takenoyama M, Hanagiri T, Oyama T, Yasumoto K (2010) Association between lymphangiogenesis/micrometastasis- and adhesion-related molecules in resected stageI NSCLC. Lung Cancer 70: 320–328 20363046 10.1016/j.lungcan.2010.02.013

[bib8] Zha J, Lackner MR (2010) Targeting the insulin-like growth factor receptor-1R pathway for cancer therapy. Clin Cancer Res 16: 2512–2517 20388853 10.1158/1078-0432.CCR-09-2232

